# Nutrient and carbon partitioning and performance for swine manure management technologies

**DOI:** 10.1002/jeq2.70223

**Published:** 2026-07-19

**Authors:** Erin L. Cortus, Mahmoud Sharara, Noelle Cielito Soriano, Kristina Jones, Daniel S. Andersen, Brett C. Ramirez

**Affiliations:** ^1^ Bioproducts and Biosystems Engineering University of Minnesota Saint Paul Minnesota USA; ^2^ Biological and Agricultural Engineering North Carolina State University Raleigh North Carolina USA; ^3^ Agricultural and Biosystems Engineering Iowa State University Ames Iowa USA

## Abstract

Swine manure management systems (MMSs) are critical for nutrient cycling. Common swine MMS storage types in the United States are deep pits, anaerobic lagoons, and slurry storages. This study presents a synthesis of literature documenting nutrient and carbon (C) partitioning via a material mass balance approach for four manure treatment technologies: acidification (ACID; *n* = 4 papers), aeration (AER; *n* = 16), solid‐liquid separation (SLS; *n* = 11), and anaerobic digestion (AD; *n* = 23) that can be used in‐line with one or more of these common storage types. Mass balance data were primarily extracted for nitrogen (N), phosphorus (P), and C (AD only) from lab and pilot‐scale studies, with nutrient flows normalized to influent characteristics. The ACID papers suggested significant reductions in gaseous losses with ACID; however, the effluent concentration and available mass/volume and gas data together did not document realistic N partitioning. Among SLS systems, urine‐feces segregation and filtration retained the most and least amount of solids in the solid fraction, respectively. AD converts organic matter into biogas; for most mesophilic digesters, 56%–66% of the C remained in the effluent; the mass retained in the sludge is underreported. In AER studies, the partitioning of N into different N gases depended on the amount of AER. As pass‐through technologies, AD and SLS are easier to incorporate in lagoon and slurry storage systems. ACID and AER can be physically incorporated in common MMSs; however, treatment could hinder anaerobic lagoon function. Understanding nutrient partitioning will benefit from concurrent monitoring of multiple nutrients in the liquid, solid, and/or gas streams.

## Plain Language Summary

Four mature technologies that can alter manure nutrient cycling are acidification, aeration, solid‐liquid separation, and anaerobic digestion. This review summarizes how well existing literature describes how each technology affects the distribution of nitrogen, phosphorus, and carbon between inputs and outputs. Acidification and aeration can reduce emissions, but it is challenging to measure both the liquid and gas streams with great certainty. Solid‐liquid separation helps concentrate nutrients into liquid and solid streams, so it is easier to compare partitioning between different types of separators. There has been considerable data collected for carbon partitioning with anaerobic digesters, and more data for other nutrients is desired. Digesters and separators are easier to incorporate in existing lagoon or slurry storage systems. Acidification and aeration can be physically incorporated into most liquid manure storage systems but may disrupt anaerobic microbial activity.

**Abbreviations**
ACID: acidification
AD: anaerobic digestion
AER: aeration
ASBR: anaerobic sequencing batch reactor
BOD: biochemical oxygen demand
COD: chemical oxygen demand
CSTR
: continuous‐stirred tank reactor
GHG: greenhouse gas
HRT: hydraulic retention time
LCA: life cycle assessment
MMS: manure management system
MSE: mass separation efficiency
OLR: organic loading rate
PAM: polyacrylamide
SE: separation efficiency
SLS: solid‐liquid separation
TAN: total ammoniacal nitrogen
TKN: total Kjeldahl nitrogen
TS: total solids
VS: volatile solids

## 1 INTRODUCTION

Manure is a product of swine production that must be managed to cycle valuable nutrients back into beneficial use and avoid environmental losses. Awareness of the environmental impacts has prompted the transformation of manure handling, storage, and treatment to reduce the effects over time (Key et al., 2015; Thoma et al., 2016). Changes in the food animal industry, including scale and regional distribution, have further influenced manure management priorities, practices, and impacts (McCann et al., 2005; Sharara et al., 2022). In recent years, the economic and environmental values of manure nutrients have become better recognized and researched (Liu & Wang, 2020). Thus, manure processing and management technologies often target one or more of the following priorities: mitigating gaseous emissions (Ndegwa et al., 2008; Sommer et al., 2013), developing multiple manure streams with different nutrient profiles (Burton, 2006; Szogi et al., 2015), optimizing manure delivery to soils (Kleinman et al., 2018), and recovering value‐added products such as fuel and energy carriers (Muhammad Nasir et al., 2012). Achievement of one or more of these priorities requires a detailed understanding of the mechanisms involved in each stage of manure management.

A swine manure management system (MMS) starts with the deposition of urine and feces and ends with the utilization of the manure product (Liu & Wang, 2020). Storage enables manure application at a time of year when beneficial for growing crops (USDA NRCS, 2026). Common swine MMS storage types in the United States are deep pits, anaerobic lagoons, and slurry storages (Thoma et al., 2016). Within the MMS and typically before or in conjunction with storage, additional treatment technologies can alter the physical and nutrient characteristics of the manure and the potential end uses. Many manure management technologies, such as anaerobic digestion (AD), solid‐liquid separation (SLS), or composting, are used in other livestock, poultry, and waste management systems (Bernal et al., 2009; Hjorth et al., 2011; Sakar et al., 2009), and their performance is dependent on process‐specific factors. Some technologies may be stacked in series (i.e., SLS before or after digestion), wherein the effluent from one treatment may be the influent for another (Hou et al., 2017). Khoshnevisan et al. (2021) cite AD as the most mature technology but recognize economic and operational challenges, particularly for developing countries.

Holistic assessment of technological intervention is therefore critical to minimize unintended consequences to management changes and to account for local constraints, economics, and farming practices while respecting individual farm settings and geography (Varma et al., 2021). Such holistic assessments use frameworks such as techno‐economic assessment, life cycle assessment (LCA), and circularity indices (van Loon et al., 2023) to capture the varied impacts of management change up and downstream of the MMS. In the process, context‐specific details such as regional crop land availability and regulatory frameworks play an important role in the adoption and success of technological interventions. These assessment techniques have different data needs in order to assess or compare technologies for nutrient flows in gas, liquid, and/or solid forms.

Manure processing and management studies, however, have not been fully standardized in terms of protocols, data collection, or scale, limiting the opportunities for statistical meta‐analysis to rank parameters within or between technologies (Varma et al., 2021). This gap creates a challenge to integrating MMS technological choices into holistic assessment frameworks. Therefore, there is a critical need to provide a systematic review and synthesis of current literature to lead toward the development of improved standardization of manure processing and management studies. The Law of the Conservation of Mass is the basic tenet behind mass transfer through control volumes of all scales, wherein “the rate at which the mass of some species enters a control volume minus the rate, which this species leaves the control volume must equal the rate at which the species mass is stored in the control volume” (Incropera & DeWitt, 1996). A material mass balance is a means to recognize all output forms for manure passing through a technology. When data for one output stream are not available, the mass flow for the missing stream can theoretically be calculated. Partitioning factors are a simplified means for expressing an output stream relative to the input stream; potential transformations of nutrients must also be considered and may be wrapped into the overall material mass balance.

Starting with a landscape survey of the state of the research for each technology, this work investigated material mass balances applied to manure treatment methods for common forms of swine MMS in the United States. Selected manure treatment technologies for this review included aeration (AER), acidification (ACID), SLS, and AD. These technologies were selected for this review because they represent a majority of manure treatment technologies applied, or applicable, at the farm scale, primarily in the United States, for liquid swine manure (Liu et al., 2014; Varma et al., 2021). Solid manure treatments like drying and composting may be viable (Varma et al., 2021) but are considered further downstream processes relative to the treatment technologies considered in this study for predominant US farm MMSs. Specifically, this review paper aims were as follows.

1. Survey and compile literature that supports material mass balances for mature processing technologies that partition and transform on‐farm manure nutrients (including carbon [C]).
2. Qualify and quantify (where possible) manure nutrient partitioning and transformations relative to influent nutrient mass for each processing technology.
3. Assess compatibility and impacts of adopting manure processing technologies on dominant US swine farm MMSs.

Each technology was investigated individually, with partitioning described relative to the input stream. The scope of this study extends from manure at the point of excretion to the output from the technology under examination. Outside the scope of this study were capital and operating costs and energy and resource use for the construction and operation of technology‐related equipment. This review is not intended as an exhaustive review of the technologies themselves; rather, a review of the ability to simplify nutrient partitioning and transformation descriptions using material mass balances with existing literature. In doing so, this review is meant to inform future research efforts and the ability to holistically model MMSs.

## 2 METHODS

In this study, four technologies for swine manure treatment were targeted in a literature review to examine nutrient partitioning and performance. The sequential steps of the process to survey, quantify, and qualify impacts for each technology were as follows: literature search → screening → tabulation → extraction and summarization → implications. Each step was iterated as needed to refine terms, processes, and internal review.

### 2.1 Literature search and screening

The literature search was conducted using the Clarivate (Web of Science) platform. Exact search terms used for each technology search are shown in Table A1. For each returned result in the literature search, the abstract was screened for relevance to this literature review using the following acceptance criteria. Additional screening occurred in the first review of each full paper.

1. A peer‐reviewed publication in the English language.
2. The abstract explicitly noted nutrient balance or nutrient fates for swine manure within a standalone technology. A sequential combination of technologies was acceptable, but only if the impact of the specific technology was potentially available (based on the abstract).
3. The paper included primary experimental data. Review and modeling papers were excluded.
4. The study provided data to quantify nutrient partitioning in all, or all but one, end products relative to the incoming manure.

There were additional technology‐specific acceptance and rejection criteria.

1. In AD studies, assay and batch testing to determine biomethane potential or specific methanogenic activity were excluded. Studies focused on startup performance of a digester or the comparison of startup methods were excluded if there was no reporting of steady‐state reactor performance after startup.
2. For inclusion in co‐digestion analyses, there was a requirement of swine manure inclusion at 50% or more of the feed (volume, mass, total solids [TS], or volatile solids [VS]). Similarly, swine manure used solely as an inoculum was not sufficient. No minimum rate of addition was imposed on codigestate feedstock. Biochars and other highly available sources of C were considered co‐digestion ingredients.
3. In the SLS technology search, studies reporting on fouling in membranes or implementation of reverse osmosis and nanofiltration were excluded. Similarly, studies reporting solely on flocculation of products and biomass, and not swine manure, were excluded.

The papers accepted during the literature review screening process were reviewed, and then information regarding scale, geographical location, and technology‐specific categories as descriptors of the process was tabulated.

### 2.2 Data extraction and summarization

Considering the placement of manure treatment before or in conjunction with manure storage, the input stream to any of the technologies would generally be as‐excreted manure or flushed manure. However, recognizing that treatments may be stacked, a general term of “influent” was assigned to the stream entering the treatment, and the influent characteristics noted. For this reason, where possible, the resulting output and storage streams were expressed as a percentage of the influent nutrient flow. The nomenclature for output and storage streams is specified in the respective Results and Discussion section for each technology.

The available nutrient information from relevant studies was extracted from tabulated and graphical data reported in the study. Graphical data were extracted using plot digitizer software (plotdigitizer.com; PORBITAL). The flows and storage amounts are presented “as‐reported” in the literature. In cases where all but one output streams’ data were presented in the literature, the missing stream was calculated by difference, assuming a steady‐state mass balance for the total mass or a specific nutrient; the output streams for each technology type are shared in the results section for each technology. In instances where mass flow data was available for a nonvolatile component like ash or phosphorus, a mass flow rate correction was compared to as‐reported data. A mass‐flow correction has been applied in barn scale studies of poultry and dairy using nonvolatile components to account for hard‐to‐measure streams like manure mass (De Boer & Wiersma, 2021; Keener & Zhao, 2008).

Separation efficiency (SE) for various nutrients was used to track fate as a result of SLS. We identified three cases wherein sufficient information is present in the literature to extract SE for nutrients of interest:

1. *Case 1*: A SE for constituent *x* (nutrient) is directly reported (SE*_x_*).
2. *Case 2*: The total mass of influent slurry (*Q*) entering the separator as well as the wet mass of separated solid fraction (*U*) are reported, in addition to constituent *x* wet‐basis concentrations (*C*) reported for both influent (IN) slurry and separated solid fraction (SF). The SE is calculated using Equation (1).
(1) $$SE_{x}=\frac{U\;\times \;C_{x.\mathrm{SF}}}{Q\;\times \;C_{x,\mathrm{IN}}}$$
3. *Case 3*: Wet‐basis concentration of TS are reported in the influent slurry, as well as in the separated solid fraction and in the liquid fraction (LF) (Equation 2), in addition to constituent *x* wet‐basis concentration in the influent slurry and separated solid fraction (Equation 3). The underlying assumption used to estimate mass separation efficiency (MSE) here is that the TS is a proxy for the total mass.
(2) $$\frac{U}{Q}\approx \mathrm{MSE}=\frac{C_{\mathrm{TS},\mathrm{IN}}-C_{\mathrm{TS},\mathrm{LF}}}{C_{\mathrm{TS},\mathrm{SF}}-C_{\mathrm{TS},\mathrm{LF}}}$$
(3) $$SE_{x}=\mathrm{MSE}\;\times \;\frac{C_{x,\mathrm{SF}}}{C_{x,\mathrm{IN}}}$$

For all technologies, nonvolatile components, like ash, phosphorus (P), potassium (K), and other micronutrients, gaseous emissions were assumed to be zero. This assumption was reviewed during the interpretation of mass balances.

Simple summaries are presented for the various output streams and nutrients with each technology where data were available. There was no attempt to balance the number of studies analyzed for each technology or nutrient, nor to explicitly estimate experimental uncertainty. Steady‐state mass balances were inferred and investigated with respect to the tabulated studies, as well as the broader literature base. The heterogeneity of experimental scales, approaches, and analytical methods is acknowledged and discussed in terms of future research needs.

### 2.3 Implications for US MMSs

For each technology, the authors present a perspective of how the technology integrates with common US swine MMSs, or base case scenarios, drawing from the broader literature and experience. In 2015, 64% of MMS in US swine production were deep‐pit manure storage, 32% anaerobic lagoons, and 4% solid storage (Thoma et al., 2016). Thus, this paper focused on technologies applicable to liquid or slurry manure storage systems, namely, deep pit, anaerobic lagoon, and slurry manure storage. Pigs are commonly housed together based on phase of production, and manure characteristics can vary accordingly (Janni & Cortus, 2020; MWPS, 2001). However, manure nutrient composition and treatment performance are both inherently variable due to a broader range of biological, dietary, management, and environmental factors. As a result, our approach was to normalize treatment performance relative to influent characteristics. The perspective for technology integration in US MMSs considers how the climate, indoor and outdoor air quality, environmental emissions, and capital and operating expenditure factor into its integration. The method of manure collection before storage especially influences the opportunities for treatment integration.

Brief overviews of the three MMSs presented in this section are derived from multiple resources (Janni & Cortus, 2020; MWPS, 2001).

#### 2.3.1 Deep‐pit manure storage system

This system consists of a concrete storage structure, varying in depth from 1.8 to 3.7 m, directly beneath the animal rearing areas within barns. Manure, feed and water spillage, and any cleaning water pass through a slatted floor into these pits. Thus, collection and storage are combined. Manure is removed from deep pits once or twice each year for land application. During these manure removal events, the pits may be agitated to suspend settled solids and are typically emptied to an unpumpable level. These systems are common to US Midwest and corn belt states, including Iowa, Minnesota, Illinois, and Indiana. MWPS (2001) suggests that 15%–30% of the nitrogen (N) is lost from this type of system between excretion and land application, presumably through gaseous losses. In the Intergovernmental Panel on Climate Change (IPCC) report, Dong et al. (2006) estimated losses of methane (CH_4_) during manure storage as 5.2 kg CH_4_ per finishing pig space per year, or about 17% of the CH_4_ potential added.

#### 2.3.2 Anaerobic lagoon storage

This system consists of an open‐air impoundment designed to provide both storage and treatment for manure. Manure is typically flushed or scraped from shallow pits beneath slatted‐barn floors and conveyed via buried piping to anaerobic lagoons. The lagoon is designed and operated to never be completely emptied, except upon decommissioning, to provide continuous treatment of manure organic matter by anaerobic microbes. Compared to deep‐pit systems, manure is removed from a lagoon in two forms: a dilute liquid “supernatant” and a high‐solids slurry “sludge.” Lagoon supernatant is land applied multiple times per year, often using irrigation equipment, whereas sludge is removed once every 5 years or more. Manure in anaerobic lagoons retains less N and C and is impacted by precipitation and evaporation. These systems are common to warmer US states, including North Carolina, Missouri, and Oklahoma, where there is lower risk of freezing weather to lagoon bacteria. MWPS (2001) suggests 70%–85% of the N is lost from this type of system between excretion and land application, presumably through gaseous loss. In the IPCC report, Dong et al. (2006) estimated losses of CH_4_ during manure storage are 20.6 kg CH_4_ per finishing pig space per year, or about 66%–78% of the CH_4_ potential added.

#### 2.3.3 Slurry storage

This system consists of an open‐air impoundment designed for storage. Such storages can be fiberglass‐lined steel tanks, in‐ground or aboveground concrete storages, or in‐ground earthen storages (basins) with compacted clay or synthetic liners. Such systems are differentiated from deep pits in that they are not located directly below the animal production zone. They are smaller than anaerobic lagoons because they do not require a constant treatment volume. Manure is typically conveyed from barns to slurry manure storages from flushed or scraped shallow pits beneath the barn via pipes. A slurry store is designed for complete emptying, with the exception of an unpumpable volume. Manure may be agitated prior to removal, once or twice per year. Manure in slurry manure storages typically retains similar amounts of N and C as a deep‐pit manure storage, but due to its uncovered construction, it is impacted by precipitation and evaporation. These systems are common in the US Midwest and corn belt states; however, current regulatory requirements in many of these states favor deep‐pit systems, wherein runoff and gaseous losses are reduced. MWPS (2001) suggested 10%– 40% of the N is lost from this type of system between excretion and land application, presumably through gaseous losses, and estimated CH_4_ emissions are similar to those for deep‐pit manure storages (Dong et al., 2006).

## 3 RESULTS AND DISCUSSION

### 3.1 Acidification

#### 3.1.1 Literature tabulation

The literature search criteria (Table A1) yielded 213 papers. The screening process using only titles and abstracts reduced the yield to 15 papers, and review of the full papers further reduced the list to four papers, per the review criteria. A metadata summary of the four papers that accommodate mass balance calculations is shown in Table 1.

**TABLE 1:** Meta‐data summary of literature supporting mass balance assessments of one or more nutrients with manure acidification treatment.

Source	Study goal	Location	Study scale	Mass balance suitability	Study duration (day)	Acid type
Wang et al. (2014)	Gas emissions based on manure type	China	Pilot	N, P	95	Sulfuric acid
Petersen et al. (2014)	Gas emissions	Denmark	Pilot	N	83	Sulfuric acid
Gioelli et al. (2016)	Gas emissions based on manure type	Italy	Lab	N	30	Sulfur powder
Regueiro et al. (2016)	Gas emissions based on acid type	Portugal	Lab	N, P	60	Sulfuric acid, alum, acetic acid, lactic acid, citric acid

Sulfuric acid or sulfur powder was used in all studies (Table 1). L. Regueiro et al. (2012) compared five acid treatments: sulfuric acid, alum, acetic acid, lactic acid, and citric acid. The amount of acid, or dosage, was reported in varying fashions. Wang et al. (2014) simply reported that acid was added to achieve a specific pH (3.5–6.5) without mentioning specific amounts. Additions were also specified as mass percentages (Gioelli et al., 2016; Petersen et al., 2014) or equivalents (L. Regueiro et al., 2012). Methodologically, a target pH approach compensates for buffer capacities within manure, which are strong between pH 4 and 7 (Sommer & Husted, 1995), and facilitates investigation of ACID mechanisms, whereas mass‐ or equivalent‐based additions support comparison of acid treatment types. In the broader literature base, Dalby et al. (2022) used nitric acid in comparison to sulfuric acid, and Piveteau et al. (2017) used sucrose to promote lactic acid formation as a form of biological ACID.

Lab and pilot scale tests are used in Table 1 papers and the majority of screened literature. Lab‐scale experiments used 0.25–5 L test vessels. Pilot‐scale experiments used 100–6500 L test vessels. The lab and pilot scale studies did not include repeated additions of fresh manure or acid. In the broader literature, there was research with full‐scale systems (Lemes et al., 2022; Overmeyer et al., 2023; Vechi et al., 2022). Overmeyer et al. (2023) used a pilot‐scale vessel (750 L) to acidify manure before returning the manure to the full‐scale pig barn's slurry channel; thus, this was considered a full‐scale experiment. Dalby et al. (2022) did simulate partial removal of manure from the lab‐scale storage. The translation of results to active storage manure vessels where manure (and other potential additions like precipitation) is continually added is limited.

Table 1 papers encompass research conducted in Europe and China. The broader literature did encompass work in Canada (Massé et al., 2015), but there were no screened papers from the United States.

Within the tabulated literature the research foci were primarily on emission reduction quantification, with consideration of manure composition post‐ACID. These data types are supportive of mass balances. In the broader literature, some ACID research (Corona et al., 2021; Piveteau et al., 2017) focused on struvite recovery.

In summary, swine manure ACID is frequently mentioned in the literature; however, the number of studies that supported mass balance investigation was few (Table 1), and the studies were primarily lab or pilot scale, with an emphasis on N and P, and made use of sulfur‐based acids.

#### 3.1.2 Extracted data summary

The mass‐balance approach hypothesis guiding data extraction was the inclusion of four streams, namely:

1. influent;
2. acid input;
3. change in stored manure over elapsed time or manure output after elapsed time—described as effluent for purposes of analysis; and
4. gaseous emissions over elapsed time.

The effluent was assumed a well‐mixed volume at the time of sampling. In actuality, during storage, there may be settling of solids in a storage that creates layers and different properties therein that may influence gas production and release. Energy input was ignored, but it is recognized that acid generation and delivery have energy requirements.

There were only four papers with sufficient data to extract or calculate mass balances (Table 2). The papers typically reported dry matter (TS), pH, VS or ash, total nitrogen (TN), and NH_4_‐N concentrations for influent and effluent characteristics. With TS, ash could be determined from a VS measurement and vice versa. Gaseous ammonia was reported in all four papers, at least for estimating emission reduction. There were reported greenhouse gas (GHG) emission measurements in two papers (Gioelli et al., 2016; Wang et al., 2014), but the units of presentation per unit surface area for any gas can prevent translation to cumulative mass losses if there is inadequate experimental setup description. This also hampered the interpretation of hydrogen sulfide data in Wang et al. (2014). Total P in influent and effluent samples was reported in half the papers.

**TABLE 2:** Total nitrogen (TN) and phosphorus (TP) mass in effluent and gaseous streams relative to influent mass for select papers that document impacts of acidification on swine manure, based on reported^a^ and ash‐adjusted^b^ total effluent mass.

Source	Slurry type	Approach	% Relative to total influent nutrient plus acid				% reduction in NH_3_ emissions	% Reduction in greenhouse gases
			TN effluent	NH_4_‐N total gaseous	N_2_O‐N total gaseous	TP effluent		
Gioelli et al. (2016)	Raw, solid w/o acid	Reported	169	6.34	1.71			
		Ash‐Adj	90					
	Raw, solid w/acid	Reported	309	2.46	0.3		65	78
		Ash‐Adj	253					
	Digested, solid w/o acid	Reported	180	19.2	2.3			
		Ash‐Adj	78.6					
	Digested, solid w/acid	Reported	272	6.55	1.8		67	61
		Ash‐Adj	148					
Wang et al. (2014)	Digestate	Reported	48.1			31.6		
	Digestate w/acid	Reported	56.8			39.3	40.2	80.8
Petersen et al. (2014)	Raw slurry w/o acid	Reported	90.8	5.1				
		Ash‐Adj	58.6					
	Raw w/acid	Reported	71.8	1.4			49–84	>90 (CH4 only)
		Ash‐Adj	46.6					
Regueiro et al. (2016)	Raw slurry w/o acid	Reported	91.7	18.0		100		
	Raw slurry w/acid	Reported	126	12.6		100	75–100	

*Note*: Reported ammonia and greenhouse gas emission reductions in select papers for acid‐treated manure or digestate relative to no acid treatment. Cells that are empty indicates that no data were available.

^a^ Reported approach assumed negligible change in mass or volume between influent and effluent streams.

^b^ Ash‐adjusted approach adjusted the effluent stream mass to equate the effluent ash mass (final mass × final concentration) to the influent ash mass (initial mass × initial concentration).

If the acid addition is considered negligible, and the mass of manure or volume of manure is assumed constant between the start and end of the experiment (negligible mass of water and other gases lost), the nutrient concentrations of the influent and effluent are sufficient for a partial mass balance, with the imbalance assumed to be gaseous losses for volatile forms of N and C. The papers reviewed reported initial mass or volume, and Wang et al. (2014) reported the final volume. However, a general observation of data from three of the four papers was a higher dry matter content of the effluent relative to the influent. Two potential causes are evaporation of water and dry matter added with the acid treatment. For example, in Gioelli et al. (2016), the effluent in the no treatment case had 50% higher dry matter content than the influent (suggesting non‐negligible evaporation), and the ratio increased further with increasing acid additions. To compensate for evaporation or account for non‐negligible acid addition, a mass balance of a nonvolatile element can be used to rectify the final volume or mass of total manure. In Table 1, mass correction, assuming ash conservation, was considered to estimate the final total volume/mass and referred to as “ash‐adjusted.” In the instance ash data were not available (i.e., I. Regueiro et al., 2016), P concentrations were considered. Methodologically, a caution for mass flow correction using low (<5% w.b) dry matter/ash samples. In a dairy farm study, mass flow correction was based on combined errors of P and potassium (De Boer & Wiersma, 2021).

While comparative studies like those in Table 2 show significant reductions in gaseous losses with acid addition, the reported effluent concentrations and available manure mass/volume data and gas data together did not result in realistic N partitioning. When the reported emissions were expressed relative to total N of the influent, the gaseous NH_4_‐N loss was 6%–19% without ACID and <1%–7% with ACID, depending on the influent and treatment conditions. Ten Hoeve et al. (2016) proposed emission coefficients for acidified manure for LCA purposes. Ammonia‐N emission coefficients were 0.064 and 0.002 kg NH_3_‐N per kg mineral N, for in‐house and during outdoor storage (with cover), respectively, in agreement with the range of results in Table 2. Hou et al. (2017) suggest 61% and 87% effluent N is retained in manure relative to influent N, considering all manure and that with ACID, respectively, in European data. Based on reported liquid concentrations and manure mass or volume (as reported and with ash adjustments), a steady‐state mass balance would suggest negative gaseous N losses in seven of the 16 cases in Table 2, which are not realistic. Ammonia loss is a function of NH_4_‐N concentration, pH, and environmental conditions (Ni, 1999). Three differences to consider among the four papers (Tables 1 and 2) are the influent material (raw liquid or solid swine manure vs. liquid or solid digested manure), duration of storage (30–95 days) and the amount of acid addition. Influent C content was not specifically reported; thus, CH_4_ and carbon dioxide (CO_2_) emissions were not reported relative to influent C in the papers. At best, one could assume influent C was equal to 55% of the influent VS (Adams et al., 1951). Per Ten Hoeve et al. (2016), CH_4_ emission coefficients were 0.002 and 0.0004 kg CH_4_‐C per kg OM for acid application in house (6‐week storage interval) or during outdoor storage, respectively. These losses represent a small fraction of influent C and would be difficult to discern from a mass balance.

In manure, P is assumed nonvolatile, though the soluble forms of P relative to insoluble forms are expected to increase with a decrease in pH (J. Zhu et al., 2006). In I. Regueiro et al. (2016), total P was completely retained (as hypothesized). In Wang et al. (2014), the ratio of effluent to influent concentrations was 32%–45%; the deviation from 100% may be related to an increase in pH promoting the formation of insoluble phosphate compounds that could be filtered out prior to effluent analysis. While more micro‐nutrient data are welcome in all future studies, sulfur should be a priority, especially when sulfuric acid is employed. There are implications for increased hydrogen sulfide formation and release, as suggested by Wang et al. (2014), and hydrogen sulfide release is a recognized danger in manure environments (Ni et al., 2018).

In summary, Table 2 data did not support quantification of volatile (N and C) nutrient partitioning in the effluent or gas streams following ACID. For nonvolatile P, the results were mixed. The application of material mass balances to ACID studies requires consideration of different measurement techniques, frequencies, and reporting units for effluent and gas streams. The acid addition is non‐negligible to overall mass balances. The degree and frequency of ACID should be considered a factor for additional refinement in future research.

#### 3.1.3 Implications for base case scenarios

Existing research has not satisfactorily addressed the frequency of acid addition for active storages receiving fresh manure daily, whether underfloor or outdoors. On‐site storage and handling of acid do need specific protocols and guidelines. The implications for emissions of land‐applied manure are not explicitly covered in this analysis, but literature supports the assumption that losses from acidified manure post‐land application are lower than non‐acidified manure (Fangueiro et al., 2015).

##### Deep‐pit manure storage

ACID is one mode of action for pit additives, which are commercially available, though desired impacts are not always realized (Duong et al., 2021). For deep pit systems, acidifiers are applied similar to other pit additives through dosing to the manure surface, often through the slatted floor. However, there remains continual addition of manure through the slatted floor and a potential need for continued dosing. The dosing frequency to accommodate fresh manure additions was not reported in the literature analyzed. There is opportunity for more sophisticated equipment to introduce and mix acid within the manure, similar to the tanker approach documented by Ten Hoeve et al. (2016), but this opportunity needs to consider risks of agitating anaerobic manure and hydrogen sulfide exposure (Ni et al., 2018). The broader literature base suggests in‐barn manure treatment offers positive implications for barn air quality and animal health and productivity (Michiels et al., 2015), in addition to reduced emissions to surroundings. Structural concerns related to acid exposure also need to be considered and addressed in longer term, full‐scale studies.

##### Anaerobic lagoon storage

ACID can be introduced to the manure at various points in a lagoon manure storage system. In‐barn surface applications are possible. Acid could be introduced during the transfer between the barn and long‐term lagoon storage, potentially improving mixing similar to Ten Hoeve et al. (2016). ACID is designed to lower pH, which would reduce the effectiveness of anaerobes and nitrifiers associated with functioning lagoons (Xu et al., 2014). Finally, acid could be introduced at the point of storage, dispersed on the surface or with a mixing system, but this would introduce risks of hydrogen sulfide exposure or microbial community impacts previously discussed.

##### Slurry storage

Similar to lagoon systems, ACID can be introduced to the manure in the barn, during the transfer, or to the storage system. Similar technical and logistical implications exist for lagoon systems. Within a slurry storage, there is less control and concern over the microbial communities compared to anaerobic lagoons, which may reduce the risks. However, slurry stores have a more concentrated effluent to treat.

### 3.2 Solid‐liquid separation

#### 3.2.1 Literature tabulation

The search terms (Table A1) used to collect SLS studies and reports for swine manure management yielded 417 studies. The abstract screening process, however, recommended 48 studies for further analysis, and a final sum of 11 studies was found to contain sufficient data for mass balance analysis and reporting (Table 3).

**TABLE 3:** Meta‐data summary of literature supporting mass balance assessments of one or more nutrients, with solid‐liquid separation treatment.

Source	Study goal	Study location	Study scale	Mass balance suitability	Separation method	Polymer type
Vu et al. (2016)	Comparison (SLS tech)	Netherland	Lab	N, P	V‐separator for urine/feces, screw press, centrifuge, flocculant (PAM), flocculant + centrifuge	PAM
Popovic et al. (2014)	Comparison (chemical/physical sep method)	Italy	Lab	N, P, K, Cu, Zn	Screw‐press, centrifuge, screw press + centrifuge	
Westerman and Bicudo (2000)	Comparison (SLS tech)	USA	Full	N, P, K, Cu, Zn	Mechanical separation (ScrewPress + screening), TFS (FeCl_3_, polymer, lime, sedimentation)	
Walker et al. (2010)	Observation	USA	Full	N, P	Roll press → flocculant‐assisted belt thickener (PAM)	PAM
Riaño and Garcia‐González (2015)	Observation	Spain	Full	N P, K, Na, Mg, Fe, Ni, Cu, Zn, Se, Mo, Cd, Ba, Pb	Screw‐press + flocculant (unknown)	PAM
Herrera et al. (2023)	Observation	Italy	Full	N, P	Screw press, vibrating mesh (0.114 mm)	
Peters et al. (2011)	Comparison	Denmark	Lab	N, P	Sedimentation, centrifuge, filtration, flocculant, coagulant + flocculant	
Pieters et al. (1999)	Comparison (SLS tech)	Belgium	Lab	N, P, K	Screw press, hydrocyclone, vibrating screen, chamber filter press, MF, RO	
K. Zhu et al. (2010)	Optimization (settling time, flocculant, dose)	Canada	Lab	P	Floc/coag + sedimentation (24 h)	
Møller et al. (2007)	Comparison (SLS method)	Denmark	Full	N, P, Mg, Cd, Pb, Hg, Ni, Cu, Zn, Cr, As	Centrifuge, screw press, floc (alum)	
Kalyuzhnyi et al. (2003)	Observation	Russia	Lab–Pilot	N, P	Packed bed filter (straw)	

Abbreviation: PAM, polyacrylamide.

Of the 48 studies that were analyzed, 21 studies solely applied mechanical separation methods (screw press, belt thickening, and centrifuge), while 17 applied a combination of mechanical and chemical separation techniques. There were 22 cases where a single separation technology was implemented with no post‐treatment, and 32 used a combination of technologies in multi‐stage separation (e.g., screw press followed by flocculation and followed by filtration). In the broader dataset, 10 papers were at full scale and deployed on a model farm. Of the full‐scale studies, all 10 contained a mechanical separation component, while only six utilized a chemical agent to assist mechanical separators.

Of the 11 studies utilized for the mass balance (Table 3), two involved US‐based research at full scale (Walker et al., 2010; Westerman & Bicudo, 2000), with the remaining studies conducted in Canada, Europe, and Asia at lab, pilot, and commercial scales. The primary mass balance indicator in SLS studies is SE. This indicator reports mass of a constituent, for example, N, P, or TS in the solid fraction (often referred to as cake or sludge) relative to its mass in the influent slurry (Møller et al., 2002; Svarovsky, 2001). In several studies, removal efficiency was reported instead of a SE to benchmark technology performance (Chastain & Vanotti, 2003; Paz Pérez‐Sangrador et al., 2012; Vanotti et al., 2002; Westerman & Bicudo, 2000). This indicator refers to change in a constituent concentration, between influent and LF, relative to its initial concentration. This indicator is often reported for water purification and filtration cases and does not provide sufficient data to estimate the mass distribution of the constituent across different fractions.

Of the 11 studies (Table 3), nine applied chemical separation methods using coagulation and/or flocculation, combined with another separation method such as filtration, sieving, and sedimentation. The common flocculants and coagulants utilized were polyacrylamide (PAM), ferrous sulfates (Fe_2_(SO_4_)_3_), ferrous chloride (FeCl_3_), and alum (Al_2_(SO_4_)_3_) (Møller et al., 2007; Riaño & García‐González, 2015; Vu et al., 2016; Westerman & Bicudo, 2000; K. Zhu et al., 2004). These articles encompass different approaches for separation, including particle‐size‐based technologies (vibrating screens, screw press, and filter press); gravitational methods (centrifuge) (Møller et al., 2007; Peters et al., 2011; Vu et al., 2016); and hydrocyclone (Pieters et al., 1999).

Many of the experiments captured in Table 3 were designed to compare SLS technologies or to document case study observations. In the broader literature, the ability to utilize part of the nutrients beyond the immediate farm land is a frequent driver for choosing SLS (Burton, 2007). Reducing the size of treatment structures, such as anaerobic digesters, was another cited driver for investigating this technology and its effect on organic matter fate (Deng et al., 2014; Woraruthai et al., 2022). Multiple studies investigated SLS as part of a multi‐step treatment strategy to produce a clean permeate for discharge to waterbodies or sewers (Carraro et al., 2024; Puchajda & Oleskiewicz, 2008). SLS goals influence the choice of separation technology.

In summary, there is a considerable body of literature (Table 3) that provides data for quantifying nutrient mass partitioning with various SLS technologies, with recognition that the mass partitioning is based on numerous measurement methods and mass partitioning estimation approaches. Some papers address multiple nutrients simultaneously, but N partitioning is the most prominent, followed by P.

#### 3.2.2 Extracted data summary

The mass‐balance approach hypothesis guiding data extraction was consideration of three streams, with the splitting of the influent stream into two effluent streams described by their solids content:

1. Influent;
2. Solids‐enriched fraction (solid fraction); and
3. Solids‐depleted fraction (LF).

Table 4 summarizes the SE for the analyzed study where mass balance was feasible. Influent slurry TS varied across studies from 0.3% (Westerman & Bicudo, 2000) to 8.5% (Vu et al., 2016), with most studies utilizing an input slurry containing between 1% and 4% TS. This TS range is reflective of the different manure handling systems typically used on farms. The most dilute slurry was associated with barn‐flushed manure, whereas the highest concentrated slurry was reflective of as‐excreted manure in which urine and feces were being segregated. Similarly, TS concentration in the solid fractions varied widely from around 4.1% TS (Westerman & Bicudo, 2000) to over 30% TS (Møller et al., 2007).

**TABLE 4:** Share of solid fraction from influent slurry under different solid‐liquid separation technologies.

Technology	Count^a^	Solid fraction share (%) of influent slurry									References
		Mass	TS	VS	TN	TP	K	Mg	Cu	Zn	
Screw press	7	8	39	70	15	16	8	16	13	12	(Herrera et al., 2023; Pieters et al., 1999; Popovic et al., 2014; Riaño & García‐González, 2015; Vu et al., 2016)
Screw press + coagulant	3	38	46		2	9	5		5		(Popovic et al., 2014)
Filtration	6	13	34	23	12	18	10	12	12	9	(Kalyuzhnyi et al., 2003; Pieters et al., 1999)
Filtration + coagulant	4	21	79	93	45	82		89	95	100	(Møller et al., 2007; Vu et al., 2016; Walker et al., 2010)
Sedimentation	1	60	96		39	61					(Peters et al., 2011)
Sedimentation + coagulant	4	20	50	118	31	26				5	(Peters et al., 2011; Pieters et al., 1999; Riaño & García‐González, 2015)
Centrifuge	6	22	47	48	30	51	84	89	33	37	(Møller et al., 2007, p. 199; Peters et al., 2011; Pieters et al., 1999; Popovic et al., 2014; Vu et al., 2016)
Centrifuge + coagulant	4	32	31	31	62	47	184		26	25	(Popovic et al., 2014; Vu et al., 2016);
Urine‐feces segregation	1	44	92	96	66	97					(Vu et al., 2016)

Abbreviations: TN, total nitrogen; TP, total phosphorus; TS, total solids; VS, volatile solids.

^a^ Number of observations (“Count”) for each individual nutrient within the same row may vary according to cited study reporting.

The separation efficiencies varied by technology as well as by tracked nutrients. Across assessed separation technologies, the solid fraction represented between 8% and 60% of the influent slurry, with a mean value of 18%. This attribute, *U/Q*, is not always reported in SLS studies. However, it could be estimated as MSE using wet‐basis TS concentrations in incoming and separated fraction using mass conservation principles (Equations 2 and 3). This approach was used in our current review, and it can introduce an uncertainty to MSE estimation due to the error compounding of TS concentration measurements, in addition to the measurement errors associated with other tracked constituents/nutrients.

Table 4 is an aggregation of heterogeneous studies, experimentation, and reported data types. However, among the studies, some patterns emerged. TS SE can be ranked based on the technology employed in a descending order as follows: urine‐feces segregation > sedimentation > centrifuge > screw press > filtration. Introducing a chemical aid to facilitate separation (coagulant and flocculant) improved technology performance. From the studies in Table 4, with the exception of urine‐feces segregation, an inverse relationship was observed between TS SE and TS concentration in the solid fraction. Sedimentation solid fraction showed TS concentrations between 9% and 11%, whereas TS concentration of the solid fraction from a screw press separation varied from 25% to 35% TS. The mean solid fraction percentage for other reported nutrients was within 10% and 5% of the total mass solid fraction percentage for screw press and filtration, respectively, and within 5% for total phosphorus but not TN for sedimentation. The mean solid fraction mass percentages for centrifuge, urine‐feces segregation, and coagulant addition in general resulted in inconsistent patterns between mass and other nutrient partitioning in the solid fraction.

Only one study (Walker et al., 2010) reported volumetric separation ratios for the solid and LFs. While underreported, this particular attribute is important for design and implementation of SLS as it informs planning storage capacity for an additional stream, that is, solid fraction, generated in the course of separation, and a recommendation for future research.

As a pass‐through process without storage within the treatment technology, gaseous losses are typically ignored; examples from the broader literature base are Aguirre‐Villegas et al. (2019) and Støckler et al. (2025). Through solid, nutrient, and C partitioning, air quality impacts can be realized when the solid or liquid effluent streams are subsequently stored or undergo additional treatment. For example, SLS can reduce GHG emissions compared to a baseline of slurry storage. However, poor management of solid stacks can increase GHG or remove benefits. The fate of the solid stream is not addressed by any of the technologies investigated in this paper, but it is recognized that solids may be directly land‐applied or candidate feedstock for further processing.

In summary, the aggregated nutrient partitioning data (Table 4) is more abundant for more nutrients and specific to SLS subset configurations, compared to ACID, AD, and AER. Table 4 provides simple estimates of nutrient partitioning to the solid fraction that appear technology subset dependent, but this analysis will benefit from closer consideration of measurement methods and within‐ and between‐study variation.

#### 3.2.3 Implications for base case scenarios

An SLS system is a viable candidate for stacking with digesters—before or after the digestion process for one or more of the influent or effluent streams. Similarly, implementing SLS prior to AER or ACID can reduce energy and/or acid inputs required to meet treatment goals. In addition, SLS has been considered for and adopted to facilitate the aggregation of nutrient‐laden fractions for further processing and conversion into value‐added products (fuels, nutrients, etc.). Mechanical separation methods require additional storage space for the solid fraction stream and the equipment installation.

##### Deep‐pit manure storage

The broad expanse of slotted floor collecting and transferring manure below grade in a deep pit system does not provide a concentrated collection point to transfer all manure through SLS. An alternative is a system that would extract manure from the deep pit storage, run the manure through SLS with return to the existing deep pit by one stream (likely the liquid stream), and separate storage for the solid stream outside the barn. Agitating deep‐pit manure for this purpose increases risk to animals and workers of hydrogen sulfide exposure, although continuous operation may reduce this risk and introduce air quality benefits (Ni et al., 2018). However, there may be potential for short‐term operation of SLS during manure agitation and removal, altering the source material immediately prior to land application.

##### Anaerobic lagoon storage

SLS systems can be readily integrated in conjunction with anaerobic lagoons to control the influent loading rate. In a multi‐stage lagoon system, solid settling basins or sedimentation may be the first stage—resulting in a height‐wise gradient of solids in the basin. Implementing an SLS step can reduce GHG emissions associated with anaerobic lagoon storage and treatment, as well as reduce the rate of sludge accumulation in the lagoon.

##### Slurry storage

For slurry storage, an SLS system could be incorporated during the transfer from barn to storage. Similar to lagoons, this may be in the form of gravity‐settling or mechanical means. For mechanical separation, additional storage space is required.

### 3.3 Anaerobic digestion

#### 3.3.1 Literature tabulation

AD literature was investigated using two searches (Table A1): one with swine manure as the sole feedstock (mono‐digestion) and the other searched studies with swine manure as a digestion ingredient (co‐digestion). Of the 499 studies identified for mono‐digestion, 55 studies were initially accepted based on information available in the title and abstract. An additional 39 studies were initially accepted as co‐digestion publications from the second search. In total, 16 and 7 studies, for mono‐ and co‐digestion, respectively, were detailed with metadata in Tables 5 and 6.

**TABLE 5:** Meta‐data summary of literature supporting mass balance assessments of one or more nutrients with swine manure anaerobic digestion treatment.

Source	Study location	Study goal	Study scale	Mass balance suitability	Digester type*	Temperature (°)	Additive(s) (if applicable)
Massé et al. (2005)	Canada	Observation	Pilot	C, N, P, K, Cu, Zn, Ca, Mg, Fe, Mn, Na	ASBR	25	
Chae et al. (2004)	South Korea	Observation	Full	C, N, P	CSTR	35	
Tao et al. (2021)	China	Comparison of metal catalysts	Lab	N, P, Zn, Cu, As	CSTR	35	Schwartmannite, Zero Valence Iron
Xiao et al. (2022)	China	Ammonia inhibition	Lab	C, N	Plug‐flow	35	Granular activated carbon
Kuglarz and Bohdziewicz (2010)	Poland	Optimization	Lab	C	CSTR	36	
Hamilton and Steele (2014)	USA	Optimization	Full	C	ASBR	22–32	
Ran et al. (2023)	China	Manure characterization	Full	C, N, P	Plug‐flow	32	
Gómez et al. (2019)	Spain	Optimization study	Pilot	C, N	Plug‐flow	34	
Massé et al. (2013)	Canada	Optimization	Pilot	C, N	Plug‐flow	40, 25, 22.5	
Hill et al. (1985)	USA	Optimization	Pilot	C, N	CSTR	55	
Lin et al. (2022)	China	Comparison	Lab	C	CSTR	37, 55, 70	
Duan et al. (2019)	China	Optimization	Lab	C	CSTR	35	
Romero‐Güiza et al. (2014)	Spain	Comparison	Lab	C, N, Cl, Na, K, Ca, Mg	CSTR	37	MgO‐ based additive
Lin et al. (2024)	China	Comparison	Lab	C	CSTR	55, 70	
Yoon et al. (2013)	Korea	Mechanisms	Lab	C, N	Plug‐flow	38	NaOCl
Schott et al. (2022)	Netherlands	P removal	Lab	P, Ca, Mg, Ca	UASB	55	Ca

Abbreviations: ASBR, anaerobic sequencing batch reactor; CSTR, continuous‐stirred tank reactor; USAB, upflow anaerobic sludge blanket.

**TABLE 6:** Meta‐data summary of literature supporting mass balance assessments of one or more nutrients with swine manure codigestion treatment.

Source	Study location	Study goal	Study scale	Mass balance suitability	Digester type*	Temperature (°)	Co‐feedstock(s) (% w/w)
Astals et al. (2012)	Spain	Optimization	Lab	C, N, F, Cl, Na, K, Ca, Mg	CSTR	Mesophilic	Crude glycerol (0, 4)
Sousa et al. (2021)	Portugal	Optimization	Lab	C, N	CSTR	36.9	Cereal and exhausted coffee liquor (0, 10, 20, 30)
Chen et al. (2021)	China	Observation (digestate recirculation)	Lab	C	CSTR	Thermophilic (digester 1), and mesophilic (digester 2)	Rice straw (25)
Azevedo et al. (2021)	Portugal	Optimization	Lab	C, N	CSTR	37	Pineapple waste (0, 20)
Panichnumsin et al. (2010)	Thailand	Feasibility	Lab	C, N, S	CSTR	37	Cassava pulp (0 – 80)
Villa et al. (2020)	Brazil	Optimization	Lab	C, N	ASBR	38	Sweet potato or cassava (0, 3, 5)
Lansing et al. (2010)	Costa Rica	Optimization	Pilot	C, N, P	Plug flow	24–36	Used cooking grease (0, 2, 5, 10)

Abbreviations: ASBR, anaerobic sequencing batch reactor; CSTR, continuous‐stirred tank reactor.

The reviewed studies were primarily conducted in China (30% of studies in Tables 5 and 6 combined) and Europe (30%). Co‐digestion was evaluated in Asia, South America, and Europe.

The scale of mono‐digestion reactors used in the surveyed studies (Table 5) varied widely. The majority of studies utilized lab‐scale (0.1–10 L) reactors, with five pilot‐scale systems (50–2500 L) and three full‐scale digesters. A sizable number of studies utilized continuous or semi‐continuous reactors that were either continuous‐stirred tank reactors (CSTRs), plug‐flow reactors, anaerobic sequencing batch reactors (ASBRs), or upflow anaerobic sludge blanket.

Co‐digestion studies reported a wide variety of feedstocks with swine manure. These included manures from other species, such as poultry and dairy, and crop residues including wheat straw, corn stover, and sugarcane bagasse. Waste generated from processing vegetables and fruits (including cabbage, beets, apples, tomatoes, sweet potato, olives, and pineapples) and industrial residues such as glycerin (glycerol), slaughterhouse waste (carcasses, innards, and keratin), and municipal solid waste have also been evaluated as co‐ingredients. All accepted studies except one (Table 6) were conducted at lab scale as batch reactors that were fed at study onset with feedstocks, in addition to the inoculum, and observed for a period between 30 and 90 days. From broader literature, it is understood that swine manure typically contains a C:N ratio of approximately 10–15. The highest biomethane yield per unit fed VS is typically observed when supplied feedstock contain a C to N ratio between 20:1 and 30:1 (Rajlakshmi et al., 2023). As such, common feedstock blends in collected studies included C‐rich feedstocks with C:N ratios exceeding 30, including coffee waste, silages, and glycerol. Ratios of swine manure to other co‐feedstocks were reported on several bases, most commonly as a percentage of total volume or masses. In some cases, feedstocks were optimized to specific mass ratios of VS (Zhong et al., 2021) or formulated to reflect specified C:N targets (Villa et al., 2020). Manure may provide buffering capacity lacking in co‐feedstocks (Ren et al., 2019). In the reviewed research (Table 6). Feedstocks were pretreated using chopping, maceration, and acid‐digestion to homogenize feedstock for AD.

Most mono‐digestion studies surveyed aimed to optimize digestion through controlling process parameters, that is, temperature, hydraulic retention time (HRT), organic loading rate (OLR), and trace element supplementation. Mesophilic digestion, that is, digestion carried out between 35°C to 40°C, was the primary studied digestion regime. However, some studies listed in Tables 5 and 6 assessed the use of additives, primarily biochar or metal catalysts, or the administration of a pretreatment such as hydrolysis hydrothermal processing as means to increase biogas productivity, immobilize metals, or mitigate the impacts of inhibitors.

In summary, a search for AD papers yielded many papers suitable for mass balance analyses that included semi‐continuous or continuous operations, albeit primarily at lab and pilot scale. Unlike ACID, SLS, and AER, there was a focus on C partitioning, with some papers also supporting N, P, and other nutrient balances.

#### 3.3.2 Extracted data summary

Mass balance streams for continuous ADs included the following:

1. Influent;
2. Effluent (digestate);
3. Gaseous product (biogas); and
4. Accumulated sludge.

Most studies contained sufficient data to facilitate C mass balances, but only after introducing assumptions regarding CC:VS and no mass accumulation in the reactor. The majority of analyzed studies focused on reporting biogas production per unit mass of VS fed or destroyed, as well as per unit mass of fed chemical oxygen demand (COD). Reported percent C in VS was used whenever reported; otherwise, VS were assumed to be 55% C by weight (Adams et al., 1951).

In AD studies, gas yields were typically reported in units of volume. Unless reported, the reported gas yields were assumed to be observed at 25°C (298.15 K) and 1 atmosphere (101.325 kPa). Under these conditions, CH_4_ and CO_2_ densities are 0.656 g/L and 1.798 g/L, respectively. Reported yields were used, along with respective gas densities, to estimate mass balance contribution of gaseous phase (biogas).

The surveyed literature (Table 7) reported between 45% and 95% of influent C is conserved in the digestate (effluent). The range of observations in mesophilic CSTRs and plug‐flow digesters was smaller, between 56% and 66%. The storage of C as sludge was assumed to be the remainder of all C after accounting for biogas production. Storage accounted for −31.5% to 81.5% of C entering the system. Even within a digester configuration, the range of C conversion efficiency captures the combined effect of process variables that influence AD, such as temperature, residence time, mixing, VS concentration in feedstock, OLR, and HRT.

**TABLE 7:** Average % nutrient in the effluent (unless noted) relative to influent nutrient mass for anaerobic digestion research.

Technology	Number of observations	% in effluent relative to influent (unless noted)										References
		C (effluent)	C (CH_4_)	C (CO_2_)	P	K	Mg	Cu	Ca	Zn	Na	
CSTR (Mesophilic)	21	65.6	21.1	9.0		96.7		0.01	83.3	0.03	95.8	(Chae et al., 2004; Duan et al., 2019; Kuglarz & Bohdziewicz, 2010; Lin et al., 2022; Romero‐Güiza et al., 2014; Tao et al., 2021)
CSTR (Thermophilic)	2	82.8	3.8	2.8								(Lin et al., 2022, 2024)
CSTR (Hyper‐thermophilic)	2	94.6	0.3	0.5								(Lin et al., 2022, 2024)
ASBR (Psychrotrophic)	3	41.5	41.6	21.4	45.7	68.9	40.5	41.6	38.4	56.5	65.1	(Hamilton & Steele, 2014; Massé et al., 2005)
Plug‐flow (Psychrotrophic)	6	50.0	29.7	15.9								(Massé et al., 2013)
Plug‐flow (Mesophilic)	25	55.5	15.7	10.0	11.4							(Gómez et al., 2019; Massé et al., 2013; Ran et al., 2023; Xiao et al., 2022)
Plug‐flow (Thermophilic)	11	64.5	20.1	14.9	30.4		22.7		30.8			(Hill et al., 1985; Schott et al., 2022)

Abbreviations: ASBR, anaerobic sequencing batch reactor; CSTR, continuous‐stirred tank reactor.

Few studies specifically discussed the mass balance of one or more nutrients. The variation in the proportion of P and other nonvolatile nutrients in effluent (Table 7) suggests effects of density and solubility on nutrient partitioning between the effluent and sludge. This gap in data reporting is explained by the primary goal of AD technology adoption, which is maximizing conversion of VS to biogas, particularly CH_4_. Aside from C and N, nutrients are assumed to be conserved in liquid and solid forms and therefore partitioned between digestate effluent and accumulated digester sludge (Muhayodin et al., 2021). Accordingly, their fate is often less prioritized in AD studies. This, however, creates a gap in understanding the transformation of volatile and agronomically relevant nutrients, such as N and sulfur.

It is a frequent practice to assume a set ratio of mass flow between influent and effluent, which could be unity or slightly below unity (no or minimal mass loss). This assumption is often used to avoid the complexity involved in measuring mass accumulation in the digester as well as the biogas mass flow. Mass accumulated in the digester during operation constitutes a large uncertainty in using a flows‐only approach. Observations of operating digesters on swine farms point to the need for routine cleanout to remove digested solids buildup (sludge). Heavy metals (Mg, Mn, Al, and Ca) are observed in much lower concentrations in reactor effluent than influent (<50% of influent) (Massé et al., 2005), with digester sludges typically enriched in these solids‐bound nutrients. These observations run in opposition to the assumption of no mass accumulation or a set rate of annual accumulation in the digester.

Reporting of N content in digester feedstocks and digestate was inconsistent. Safley and Westerman (1994) could not conclusively explain rationale for a change in concentration of total Kjeldahl nitrogen (TKN) between influent and digestate and whether loss in biogas, retention in sludge, or both are the driver. A study utilizing ASBRs for swine manure digestion reported TKN conservation during the process, despite the shift in ammonium‐to‐TKN ratio from 74% in raw manure to 85% in digestate. By contrast, several studies have reported significant retention percentages for P, Ca, Cu, Zn, and S in the digester due to natural settling. In two lab‐scale experiments (Astals et al., 2012; Sousa et al., 2021) with CSTRs under mesophilic conditions, the relative ratios of total ammoniacal nitrogen (TAN) to TKN ranged from 0.5 to 0.75 in the co‐feedstocks, while the digestate contained a TAN:TKN ratio between 0.56 and 0.88 (Astals et al., 2012; Sousa et al., 2021).

In summary, for the majority of mass‐balance data evaluated, 56%–66% of the influent C remains in the effluent; the amount in the sludge is not well‐accounted for (Table 7). While AD is considered a mature technology, additional data on N and other micronutrients will further support optimization of the digestion process and related output streams, as well as consideration of the different frequencies and methods for effluent, gas, and sludge quantification.

#### 3.3.3 Implications for base case scenarios

There are many full‐scale systems in operation in the United States (US EPA, 2025) and worldwide. Digesters may be on‐farm or serve communities of farms, with and without other feedstocks. Digestion is stackable with other technologies. In all cases, digestate characteristics would need to factor into nutrient management plans for land application.

##### Deep‐pit manure storage

Meinen et al. (2014) is an example of incorporating AD into a deep pit system. This case study included separation of raw and digested manure under the floor and the AD outside the barn. This was a significant modification. There are disease transmission (biosecurity) concerns for the introduction or reintroduction of manure back into the animal area.

##### Anaerobic lagoon storage

Swine farms containing anaerobic lagoons carry out AD as part of their operation. Uptake of AD for biogas collection by US swine farms has been the greatest for farms with this form of MMS (US EPA, 2025). This is typically accomplished either by installing covers on existing anaerobic lagoons or constructing a separate structure for enclosed AD and gas collection. In the latter case, the existing anaerobic lagoon functions as a secondary treatment and a digestate storage structure. The growing number of on‐farm ADs on swine farms that utilize anaerobic lagoons can be attributed to the existing infrastructure for manure collection that achieves the dilution level recommended for ADs. Additionally, these farms are located in the Southern Midwest or Southeast (US EPA, 2025), where weather conditions promote AD activities without the need for supplemental heat. After AD treatment, the digestate may be more prone to ammonia losses, particularly with storage in open lagoons and in warm climates.

##### Slurry storage

An AD can be installed in‐line between the barn and the slurry tank. Slurry storages can potentially be retrofitted for operation as AD, which would conserve space and leverage existing infrastructure.

### 3.4 Aeration literature tabulation

The literature search criteria (Table A1) yielded 263 papers. The screening process using only titles and abstracts reduced the yield to 37 papers. With further review, 16 papers contained sufficient data for an N (*n* = 12) or P (*n* = 7) mass balance (Table 8).

**TABLE 8:** Meta‐data summary of literature supporting mass balance assessments of N and P, with aeration treatment.

Source	Study goal	Location	Scale	Mass balance suitability	Study duration (d)	Frequency	Aeration rate (*L*_air_/*L*_manure_/min unless noted)	Air introduction	Manure addition
O'Halloran (1993)	Gas trapping	Canada	Lab	N	15	Continuous	2	Pneumatic	Batch
Mackenzie and Tomar (2011)	Comparison	USA	Lab	N	15	Cont/Int	0.0667	Pneumatic	Batch
Burton et al. (1993)	Observation	UK	Pilot	N	4, 1.5	Continuous	0.2	Pneumatic	Continuous
Burton and Farrent (1998)	Comparison	UK	Pilot	N		Continuous	0.034, 0.052, 0.026	Pneumatic	Continuous
Bicudo and Svoboda (1995)	Observation	Portugal	Full	N		Intermittent	DO = 2.5 mg/L	Mechanical	Continuous
Yang et al. (2004)	Observation	USA	Lab	P	15	Intermittent		Pneumatic	Batch
Park et al. (2005)	Comparison	USA	Lab	N	22	Continuous	0.0013, 0.003, 0.005	Pneumatic	Batch
Beline et al. (2001)	Comparison	France	Lab	N	2, 4	Continuous	DO = 3.8 mg/L	Mechanical	Continuous
Luo et al. (2002)	Comparison	USA	Lab	P	1	Continuous	0.067	Pneumatic	Batch
Wu et al. (2010)	Observation	USA	Lab	P	15	Continuous	0.129	Pneumatic	Batch
Yang and Baidoo (2005)	Comparison	USA	Lab	P	21	Continuous	0.03	Pneumatic	Batch
J. Zhu et al. (2006)	Comparison	USA	Lab	N, P	15	Continuous	0.13	Pneumatic	Batch
J. Zhu et al. (2005)	Observation	USA	Lab	N	21	Continuous	0.036, 0.085, 0.135	Pneumatic	Batch
Calvet et al. (2017)	Observation	UK	Lab	N	42	Intermittent	0.17	Pneumatic	Batch
Luo et al. (2001)	Comparison	USA	Lab	P	15	Cont/Int	0.065	Pneumatic	Batch
Béline et al. (1999)	Observation	France	Lab	N	60	Continuous	DO = 2, 4 mg/L	Mechanical	Batch

The accepted studies in Table 8 consisted of one full‐scale, three pilot‐scale, and 12 lab‐scale studies. Ten of the accepted studies were conducted in North America (United States and Canada), and the remaining six studies were conducted in Europe. Lab and pilot scale studies’ durations were <60 days; the full‐scale study (Bicudo & Svoboda, 1995) was conducted over 9 months, though different AER treatments were applied in 2–3 month increments. Of the Table 8 papers, only J. Zhu et al. (2006) provided sufficient N and P data for balance estimates, whereas all other papers focused on N or P. No papers listed in Table 8 provided sufficient data for a C balance, though changes in biochemical oxygen demand (BOD), BOD5, or COD were measurands in some studies (Bicudo & Svoboda, 1995; Park et al., 2005; J. Zhu et al., 2005). All lab‐scale and one pilot‐scale study were conducted as batch experiments; the remaining two pilot‐scale and full‐scale studies considered continuous addition of fresh manure.

The modes and durations of AER were highly variable, and AER rates were reported using several units across studies. Comparable AER rates were not available for three papers that solely reported design dissolved oxygen levels of 2.5 mg/L (Bicudo & Svoboda, 1995), 3.8 mg/L (Beline et al., 2001) and 2–4 mg/L (Béline et al., 1999). For the remaining studies in Table 8, AER rates were converted into a common unit of *L*_air_/*L*_manure_/min, with the following assumptions:

1. Swine manure density is equivalent to water (1000 g/L).
2. Air is made up of 21% O_2_.
3. Air density = 1292 mg/L.

The AER rates varied from 0.0013 to 2 *L*_air_/*L*_manure_/min, where manure volume is the reactor capacity. Influencing the AER rate was continuous or intermittent delivery of air through the manure.

AER studies primarily focused on N measurements to investigate ammonia emissions, nitrification and denitrification processes, or phosphorus forms that influence pollution effects post land application. Within these overarching goals, experiments were often designed to examine different AER rates or frequencies, with and without other chemical property influences. One paper (O'Halloran, 1993) used AER as a backdrop to create different ammonia loss rates to examine acid‐trap efficiency.

In summary, Table 8 demonstrates at least 17 studies provide data that support mass balance‐based nutrient partitioning exploration using AER. However, only one of the papers was a full‐scale demonstration with intermittent AER and continuous manure addition. Each paper focused on N or P, and rarely both simultaneously.

### 3.5 Extracted data summary

The mass‐balance approach hypothesis guiding data extraction was the inclusion of three streams for each nutrient of interest:

1. Influent;
2. Change in stored manure over elapsed time or manure output after elapsed time—shown as effluent for purposes of analysis; and
3. Gaseous emissions over elapsed time

In batch studies, initial and final manure nutrient concentration data represented the influent and effluent manure, assuming no change in total manure mass. For studies with continuous manure flow (Bicudo & Svoboda, 1995; Burton & Farrent, 1998; Burton et al., 1993), average influent and effluent concentrations were used to perform the balance with the assumption of no storage in the reactor. For the N balance, we assumed that reported TKN was equal to TN concentration. However, this assumption is not wholly recommended, as some AER conditions can result in nitrite concentrations of 33% (Burton et al., 1993) and 11% (Béline et al., 1999) of the total N in the raw slurry. Burton et al. (1993) noted use of a modified Kjeldahl method to reduce all nitrate and nitrite for inclusion in the total N measurement. The sum of both organic and inorganic P in the soluble and insoluble fractions was used to estimate total P used in the balances. For the two studies that included multiple phases, the analysis considered the phases separate (Bicudo & Svoboda, 1995) and aggregated Phases 1 and 2 (Calvet et al., 2017).

Summarized in Table 9, across papers of varying scales and analysis methods, the average effluent total N was 90% of the influent without AER. With AER, the average was 68%, suggesting 32% of the N was lost in gas form. As expected and as shown by the standard deviations, there was variation among treatments and papers. Considering all intermittent AER results, the gaseous losses were 57%. Among papers, the duration and scale of the study would contribute to the percent retention in the effluent and loss via emissions. The lowest effluent concentration (3%–20% of influent concentration) (and greatest gaseous loss, 80%–97%) was the full‐scale experiment with intermittent AER (Bicudo & Svoboda, 1995). Excluding these results suggests the average effluent concentration was 73% of the influent concentration, and moreover, the average effluent concentration with intermittent AER was 87% of the influent concentration (Table 9), with little difference from no AER. It seems plausible continuous AER would generate more gaseous losses. Interactions between other factors that may affect nitrification/denitrification (e.g., temperature) may also influence the total gaseous N losses (Béline et al., 1999). This is the case in Park et al. (2005), where authors observed that high AER rate and low temperature or low AER rate and high temperature combinations were optimal conditions for reducing NH_3_ emissions.

**TABLE 9:** Average ± standard deviation effluent (*n* = number of results) and unaccounted‐for losses among papers listed in Table 8.

	Effluent (as % of influent)		Losses (as % of influent) − estimated	
	N	P	Total N	P
No aeration^a^	90 ± 14 (*n* = 5)	98 ± 3 (*n* = 8)	10 ± 14 (*n* = 5)	2 ± 3 (*n* = 8)
With aeration^b^	68 ± 22 (*n* = 51)	94 ± 8 (*n* = 14)	32 ± 22 (*n* = 51)	6 ± 8 (*n* = 14)
Lab/pilot scale only^c^	73 ± 15 (*n* = 47)	94 ± 8 (*n* = 14)	27 ± 15 (*n* = 47)	6 ± 8 (*n* = 14)
Intermittent^d^	43 ± 42 (*n* = 7)	97 ± 3 (*n* = 3)	57 ± 42 (*n* = 7)	3 ± 3 (*n* = 3)
Lab/pilot scale only^e^	87 ± 9 (*n* = 3)	97 ± 3 (*n* = 3)	13 ± 9 (*n* = 3)	3 ± 3 (*n* = 3)
Continuous^f^	72 ± 15 (*n* = 44)	93 ± 9 (*n* = 11)	28 ± 15 (*n* = 44)	7 ± 9 (*n* = 11)

*Note*: Losses were estimated by subtraction.

^a^ (Beline et al., 2001; Dube et al., 2016; Hanhan et al., 2011; Luo et al., 2002; Martinez et al., 1995; Mostafa et al., 2019, 2020; Ndegwa, 2004; Ndegwa et al., 2003; J. Zhu et al., 2006).

^b^ All papers referenced in Table 8.

^c^ Same as b, excluding Bicudo and Svoboda (1995).

^d^ (Bicudo & Svoboda, 1995; Calvet et al., 2017; Luo et al., 2001; Mackenzie & Tomar, 1987; Yang et al., 2004).

^e^ Same as d, excluding Bicudo and Svoboda (1995).

^f^ All papers referenced in Table 8, excluding Calvet et al. (2017), Yang et al. (2004), and Bicudo and Svoboda (1995).

The studies in Table 9 provided four instances to compare effluent/influent‐based N balance estimates with corresponding gaseous loss measurements. The work by Beline et al. (2001) and Béline et al. (1999) provided N fate descriptions documenting 60%–110% recovery of influent N in effluent (organic N, ammonium, nitrate, and nitrite) and nitrous oxide and ammonia emission measurements. In a lab‐scale experiment, Beline et al. (2001) estimated 15.4% and 34.5% of total influent N was removed (assumed lost as emissions) in two AER experiments; 48%–63% of the removal (loss) was characterized as nitrous oxide and negligible loss as ammonia. Up to 30% N_2_O (as a percentage of the influent N) was reported in another study (Béline et al., 1999). Burton et al. (1993) accounted for 2.1% and 21.3% of total N losses estimated based on effluent concentrations in the forms of N_2_O, NH_3_, nitrogen oxide, and nitrogen dioxide. O'Halloran (1993) reported acid‐trap‐recovered NH_3_ could account for up to 95% of the N loss based on effluent‐influent measurements, but the trapping efficiency depended on the acid and trap configuration. A 20% increase in ammonia emissions with AER was reported by Calvet et al. (2017), but the total ammonia emission rate was 6% relative to influent N, with or without AER, and there was minimal N loss (−4% to 2%) based on effluent characteristics. The different AER schemes across studies in this work provided conditions that were conducive to varying degrees of nitrification and denitrification, affecting the relative concentrations of N_2_O, NH_3_, dinitrogen gas, and nitrogen dioxide. Dinitrogen gas is notoriously difficult to measure and is often assumed to be the unaccounted loss when other gases can be measured (Neysari et al., 2023). The environmental impact of the release of different nitrogenous gases is an important consideration when applying findings for AER to an LCA or other holistic analysis.

Since P is not volatile, a high nutrient recovery (100% ± 10%) was expected, with consideration of possible measurement errors or inconsistencies during sample collection and processing. The average effluent concentration in the analyzed studies (Table 9) was 98% and 94% with and without AER, respectively. Unlike ACID studies, evaporation did not appear to challenge the assumption of equal influent and effluent mass in batch AER experiments. Calvet et al. (2017) reported a 10% decrease in slurry volume between the start and end of the experiment; P concentrations were not reported, but the increase in ash concentration was <0.16% w.b. Typically, the transformations of P between soluble and insoluble and organic and inorganic forms were the greater interest in these papers, which all contained a common author/research group (Zhu). The mass balance approach works well when accounting for total P in the manure but does not account for transformations. For this reason, P balance results from this work may not be as useful when predicting P availability for crops during land application and should be used in conjunction with other supplementary data.

It is interesting to note that AER is an approach to transform OM into CO_2_ and other inert OM, yet there is little data on C partitioning and transformation, unlike AD.

In summary, compiled results from manure AER experiments showed material‐based mass balances using influent and effluent data suggest 43%–73% retention of N in the slurry. Ammonia emissions can be reduced, but nitrification and denitrification products deserve more attention in future research for mass balance accounting. The majority of P is conserved in the effluent, but there was a lack of data for C, K, and micronutrients. Further work is needed to better understand how AER schemes influence the fractions of different N and C gases.

### 3.6 Implications for base case scenarios

The literature reviewed demonstrated various configurations to introduce AER (mechanical and pneumatic), frequency, and mode that make this technique adaptable to treatment goals but result in inconsistent impacts. the mode and frequency of AER has bearing on decision‐making related to cost, maintenance, and installation of AER systems in existing swine manure storages. The lifespan of AER systems in the manure environment, with and without freezing weather conditions, is not well reported. Electricity consumption was not considered in this analysis. Furthermore, temperature affects solubility and nutrient fractions solubilized in the manure and may affect gas emission rates from these systems.

#### Deep‐pit manure storage

Installation and maintenance of equipment below an existing slatted floor is difficult and requires access points. Theoretically, the benefits of AER increase if the AER system can accommodate variable depths. Volatiles will still move through the animal airspace, with or without pit ventilation. However, reductions in ammonia and other anaerobically produced gases are generally considered positive for animal and worker health and productivity in the barn. Incomplete nitrification and denitrification could contribute nitrous oxide to the surroundings. Deep‐pit manure retains more consistent and above‐freezing temperatures year‐round, which may further support aerobic bacteria and gas solubility.

#### Anaerobic lagoon storage

AER is contrary to an anaerobic lagoon's purpose and mechanism but could support facultative processes. In a multistage system, AER may be applied to one or more of the stages.

#### Slurry storage

AER would provide treatment for this form of manure storage. Similar to lagoons, an AER system may be applied to one or more of the stages in a multistage system. In cooler climates, ice build‐up may inhibit equipment operation and necessitate seasonal installation and removal.

## 4 Ordering of technologies

Each technology was reviewed and analyzed as a stand‐alone technology and discussed in line with a base case MMS. In actuality, and as demonstrated in much of the literature, the technologies may be stacked with one another. By normalizing the output streams to the influent characteristics, an attempt was made to accommodate a broader range of influent characteristics. However, one must be judicious in applying the results for scenarios that lack evaluation. If the manure characteristics are a factor in the technology process, even more attention is needed.

An example stack is SLS and AD. SLS may precede AD to create a solids content amenable to the digester format (plug flow and CSTR) or proceed AD to separate solids from digestate. AER or ACID are options to reduce gaseous ammonia, specifically, from AD digestate post‐treatment while in storage. AER and ACID are less likely to proceed with AD as they work against anaerobic microorganism environments.

## 5 Limitation of approach used in this paper

A literature search for four manure management technologies yielded hundreds of papers. The number of papers that provided sufficient data for mass balance evaluation was limited to 4 for ACID, 11 for SLS, 23 for AD, and 16 for AER. Papers were never able to fully measure all streams for N and C, given the multiple forms and transformations, acknowledging that some forms (i.e., dinitrogen gas) are particularly hard to measure. Balances of nonvolatile components, like total P, were limited but were reasonably accounted for in mass‐balance exercises for SLS and AER.

The limitations of the approach used in this paper are recognized. In the literature review screening, the reduction in the number of papers is indicative of false positives based on abstract review. There is potential for false negatives related to databases searched and/or search terms and/or interpretation by the authors. There were efforts to minimize subjective differences during interpretation through multiple author review of search lists.

In the preceding analyses, data were compiled and presented to facilitate modular use in model development, that is, as a stand‐alone technology or part of a technology stack. However, the summarized data do not reflect some interactions that may arise from ordering of the technologies in a given stack. This paper does not provide estimates of uncertainty around mass balance. This is primarily due to sparseness of datasets.

## 6 Limitations of existing datasets

Each paper used in this review had specific goal(s); if mass balance data were present, it was to verify results pertinent to another goal. The mass balance approach is grounded in the Law of Conservation of Mass and theoretically validates measurements of different forms reduced to a single element. There was no shortage of papers for any given technology, but there was a surprising lack of data to support mass balances. For all technologies in this analysis, one stream was typically calculated by subtraction. In fact, we are missing dedicated mass balances for our base cases themselves, without technology additions, that have been around in broader numbers and for a longer time.

We relied on reported averages of nutrient concentrations and operational parameters. A mass balance approach does not capture conditions that may have led to more drastic, instantaneous change of nutrient partitioning in the effluent versus the air.

Studies were weighted equally, regardless of scale or location. Treatment effects at laboratory scale were assumed representative of real‐world applications for all climatic conditions. On‐farm practices and environmental conditions may vary between regions considered in this analysis, including frequency and proportion of manure removal each year. AER is a good example where drastic differences were evident between field and lab/pilot‐scale experiments.

Different methods used for gas, liquid, and solid stream measurements introduce varying amounts of uncertainty to the mass balance; this is apparent in Table 2. Manure nutrients are typically based on a sub‐sample of the overall volume, and the mass or volume may be reported periodically. Air sampling may be sporadic, semi‐continuous, or continuous. Accurate accounting of these uncertainties is needed for robust mass balance estimates.

Mass balance is often necessary (crosscutting need) for next steps in the research and development pipeline, that is, the development of process models and LCAs.

## 7 Future needs and considerations

The ability to validate a mass balance supports broader investigation of impacts via manure and air streams and demonstrates research integrity. The consideration of two or more nutrients simultaneously can strengthen the understanding of mechanisms and transformations, especially if there are measurements of both volatile and nonvolatile compounds. A minimum need and consideration is reporting the initial and final mass and/or volume and nutrient concentration(s) in batch experiments. Evaporation should not be ignored when concentrations are solely reported. The sharing of these measurements will facilitate mass balances by others, as will disclosure of underlying assumptions for intercomparability between studies.

There is a lack of long‐term and operationally realistic datasets for all four technologies. However, it is acknowledged that mass balance data collection ease (time, equipment, cost, etc.) is inversely related to MMS scale. To compensate with lab and pilot‐scale research, the authors recommend more frequent additions of ACID treatment and/or fresh manure in ACID and AER studies. When sampling frequency decreases, less can be deduced on the mechanisms that relate to dynamic factors in microbial systems like AD and AER, but it may suffice for some research questions. For mature treatment technologies like AER, ACID, SLS, and AD, the integration, longevity, and sustained performance are a research need, ideally with data to support mass balance verification.

There are limited data on nutrients other than N, C, and P. To address this gap, future research may consider whether certain nutrients (i.e., P) can serve as proxies for the partitioning of other nonvolatile nutrients post treatment. This approach should consider the solubility, potential transformations, and measurement methods of the nutrient of interest.

## AUTHOR CONTRIBUTIONS

**Erin L. Cortus**: Conceptualization; data curation; formal analysis; methodology; supervision; writing—original draft; writing—review and editing. **Mahmoud Sharara**: Conceptualization; data curation; formal analysis; methodology; supervision; writing—original draft; writing—review and editing. **Noelle Cielito Soriano**: Data curation; formal analysis; methodology; writing—original draft. **Kristina Jones**: Data curation; formal analysis; methodology; writing—original draft. **Daniel S. Andersen**: Conceptualization; methodology; writing—review and editing. **Brett C. Ramirez**: Conceptualization; methodology; writing—review and editing.

## CONFLICT OF INTEREST STATEMENT

The authors declare no conflicts of interest.

## 1

**TABLE A1:** Search terms used in literature survey for different manure processing and conversion technologies.

Technology	Search term
Solid‐liquid separation	(TI = (swine OR sow OR hog OR pig) OR AB = (swine OR sow OR hog OR pig) OR AK = (swine OR sow OR hog OR pig)) AND (TI = (solid‐liquid separation) NOT (TI = (wetland* AND pond*)) OR (AB = (solid‐liquid separation OR centrifugation OR filtration OR settl* OR floc* OR screening OR membrane)) NOT (AB = (chlortetracycline OR gas‐permeable)) OR AK = (solid‐liquid separation)) AND (TI = (manure OR slurry) OR AB = (manure OR slurry) OR AK = (manure OR slurry)) NOT (ALL = (odor OR odour)) NOT(TI = (antibiot* OR sanit* OR diversity OR indicator OR pathog* OR vir* OR coliform OR antimicrob* OR community OR surviv* OR gene* OR geno* OR microb* OR alga* OR bact*)) NOT(TI = (economic OR econ*)) NOT (AB = (soil OR runoff OR leachate)) NOT (ALL = (diet)) NOT (TI = (life cycle OR LCA)) NOT (TI = lagoon)
Acidification	(pork OR swine OR sow OR hog OR pig) AND (acidif*) NOT (antibiotic OR sanit* OR diversity OR pathog* OR viral OR coliform OR antimicrobial) AND (manure OR slurry) NOT (odor* OR odour*) NOT (economic OR econ*) NOT (diet) NOT (soil) NOT (land application)
Anaerobic mono‐digestion	(pork OR swine OR sow OR hog OR pig) AND (anaerobic digestion) NOT(antibiot* OR sanit* OR diversity OR indicator OR pathog* OR viral OR coliform OR antimicrob* OR community) AND (manure OR slurry OR wastewater) NOT (odor* OR odour*) NOT (codigestion OR co‐digestion OR coferm* OR co‐ferm*) NOT (economic OR econ*) NOT (diet) NOT (soil)
Anaerobic Co‐digestion	((TI = (pork OR swine OR sow OR hog OR pig) OR AB = (pork OR swine OR sow OR hog OR pig) OR AK = (pork OR swine OR sow OR hog OR pig)) AND (TI = (anaerob* digest*) OR AB = (anaerob* digest*) OR AK = (anaerob* digest*)) AND (TI = (manure OR slurry) OR AB = (manure OR slurry) OR AK = (manure OR slurry)) NOT (ALL = (odor OR odour)) AND (ALL = (codigest* OR co‐digest* OR coferm* OR co‐ferm* OR addit* OR combin* OR supplem*)) NOT(TI = (antibiot* OR sanit* OR diversity OR indicator OR pathog* OR viral OR coliform OR antimicrob* OR community OR surviv* OR gene* OR geno* OR microb*)) NOT(TI = (economic OR econ*)) NOT (AB = (soil OR runoff OR leachate)) NOT (ALL = (diet)) NOT (TI = (lagoon OR impermeable cover)) NOT (TI = (life cycle OR LCA)))
Aeration	(pork OR swine OR sow OR hog OR pig) AND aerat* NOT composting AND (manure OR slurry) NOT (odor* OR odour*)

Abbreviation: LCA, life cycle assessment.
